# Quantum correlations in the frustrated XY model on the honeycomb lattice

**DOI:** 10.1038/s41598-023-43080-3

**Published:** 2023-09-25

**Authors:** Sahar Satoori, Saeed Mahdavifar, Javad Vahedi

**Affiliations:** 1 Department of Physics, University of Guilan, Rasht, 45196-313 Iran; 2 School of Engineering and Science, Jacobs University, Campus Ring 1, 28759 Bremen, Germany; 3 Department of Physics, Sari Branch, Islamic Azad University, 48161-19318 Sari, Iran

**Keywords:** Condensed-matter physics, Physics, Phase transitions and critical phenomena

## Abstract

We have investigated the spin-1/2 XY frustrated antiferromagnetic Heisenberg honeycomb model, which features an intermediate region in its ground state phase diagram that is not well understood. The two dominant phases in the diagram are the quantum spin-liquid (QSL) and the antiferromagnetic Ising order. Quantum correlations suggest that the QSL phase is likely to exhibit entanglement. To explore this possibility, we utilized numerical Lanczos and density matrix renormalization group (DMRG) methods to calculate concurrence, quantum discord (QD), and entanglement entropy. The results of our study indicate the existence of quantum entanglement within the intermediate region, implying a greater probability for the dominance of the quantum spin-liquid (QSL) phase over the antiferromagnetic Ising order. This discovery underscores the importance of considering quantum correlations in comprehending the model’s behavior and provides insight into the complex nature of quantum systems.

## Introduction

The phenomenon of physical quantum phase transitions in low-dimensional magnets is a fascinating topic in condensed matter physics^1,2^. These transitions manifest themselves through quantum fluctuations even at absolute zero temperature, with their effects depending on factors such as the spin quantum number, the coordination number and the degree of frustration.

Frustrated low-dimensional spin systems play a key role in unravelling the enigmatic quantum phases of matter^3^. Magnetic frustration arises from the conflicting interactions between spins that cannot be accommodated simultaneously, leading to a state of macroscopic degeneracy at the bottom. In recent decades, a wealth of theoretical and experimental investigations have been devoted to the exploration of novel quantum phases in frustrated systems with one or two dimensions (1D and 2D). A prime example is the study of spin-1/2 antiferromagnetic isotropic frustrated Heisenberg chains, where extensive research has demonstrated a quantum phase transition from a Luttinger liquid phase to a dimer phase at a critical frustration value^4–6^.

Research into two-dimensional frustrated models has focused mainly on lattice configurations such as the triangular, square, Kagome and honeycomb arrangements^7^. One reason for studying these 2D systems is the emergence of an extraordinary quantum phase known as the quantum spin liquid (QSL)^8–10^. The unveiling of high-temperature copper oxide superconductors^11,12^ and Anderson’s report of the existence of the resonating valence bond (RVB) grand state within the antiferromagnetic Heisenberg model on a hexagonal lattice^13^ paved the way for the exploration of the QSL phase.

Among the many frustrated systems on two-dimensional lattices, particular emphasis has been placed on the spin-1/2 honeycomb lattice. In particular, in the absence of frustration, the isotropic Heisenberg honeycomb model exhibits a state of long-range Néel order^14–16^. However, in 2001, using the Lanczos numerical method, researchers identified a quantum phase transition into either the quantum spin liquid (QSL) or dimer phase induced by the presence of frustration^17^. This discovery was subsequently confirmed by various numerical and analytical techniques^18–25^. In addition, experimental studies carried out at low temperatures have suggested the candidacy of certain materials as potential QSL candidates^26,27^.

In addition to the isotropic Heisenberg model, extensive work has been done on the ground state phase diagram of the spin-1/2 frustrated honeycomb XY model. Varney and colleagues led the study of this model using the numerically exact diagonalisation method^28^. By focusing on the fidelity parameter within finite-size lattices, they discovered the presence of four distinct phases, bounded by three critical quantum phase transitions, within clusters of $N=24$ spin-1/2 particles. These phases include the Néel state, a spin wave state characterised by $120^{\circ }$ order, two intermediate phases: a quantum spin liquid (QSL) and an exotic spin wave state.

The resilience of the QSL phase to various perturbations is highlighted by the application of the numerical Lanczos method^29^. Further insights into this intermediate region are gained by employing variational Monte Carlo techniques, which show that the ordered phases lose energy to an exotic fractionalised partonic wave function, in agreement with the envisaged gapped QSL phase^30^. Moreover, the stability of the QSL in the intermediate region is supported by extended path integral Monte Carlo simulations, in contrast to the Ising phase proposals of the density matrix renormalisation group^31^. However, an alternative analytical study using a variational approach based on Jastrow wave functions refrains from confirming the existence of the QSL phase within this intermediate region^32^.

However, the use of the density matrix renormalisation group (DMRG) method for numerical investigations has brought perplexing challenges and uncertainties to the field. Instead of the expected quantum spin liquid (QSL) phase, the intermediate frustration regime reveals the emergence of an antiferromagnetic Ising phase characterised by a staggered magnetization directed along the *z*-axis^33,34^. These results are accompanied by a significantly lower ground state energy compared to that of the proposed QSL phase and a conspicuous absence of topological entanglement entropy. Further insights are gained from the application of the series expansion method, which shows that in the vicinity of the first critical point, the nearest *zz* correlations escalate to reach equivalence with the nearest *xy* correlations. This result indirectly supports the existence of the Ising phase within the intermediate region^35^. The orientation of the spins in the *z* direction within an ordered phase is confirmed by subsequent studies using coupled cluster methods^36^.

Recent investigations, exploiting the capabilities of the DMRG, explore the influence of a three-spin chiral term and establish the persistent stability of the Ising phase within the intermediate region even in the presence of such perturbations^37^. The framework of boson-vortex duality has been used to scrutinise the above model, showing that the condensation of one of the two vortex flavours corresponds to the emergence of the Ising phase in the intermediate region. Conversely, the condensation of both vortex flavours leads to the replacement of the QSL^38^. Furthermore, the field of bosonic dynamical mean-field theory has recognised the emergence of an emergent chiral spin state in the intermediate frustration regime, replacing the dominance of both the Ising and QSL phases^39^. A distinctive study based on the Chern-Simons fermionisation of the spin 1/2 operators reveals a complex structure of ground state order within the intermediate regime. This structure is characterised by the coexistence of out-of-plane long-range N’eel ordering and in-plane chiral spin-liquid ordering^40^.

**Figure 1:**
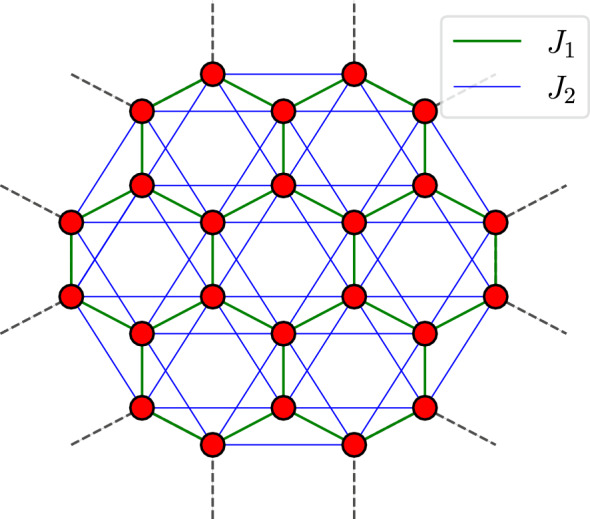
The cluster of $N=24$ spins with hexagonal symmetry. Green and blue links show coupling between nearest and next-nearest neighbor spins, respectively. A twist periodic boundary condition is implement.

In essence, the ground state phase diagram of the 2D spin-1/2 XY frustrated antiferromagnetic Heisenberg honeycomb model remains exotic and subject to controversial interpretations. This complexity is underlined by the multitude of conflicting results and perspectives from different computational approaches and theoretical frameworks. Inspired by the pivotal role of quantum correlations in identifying the critical quantum phase transition points, which encompass both symmetry and topological properties, we embark on a series of numerical analyses of the ground state phase diagram of the spin-1/2 XY frustrated honeycomb model. By delving into the concepts of quantum entanglement, we reveal a distinctive way of understanding strongly correlated systems close to their critical thresholds^41^.

In this study, we employ the numerical Lanczos and density matrix renormalisation group (DMRG) methods to perform a comprehensive investigation. We focus on the evaluation of key entanglement measures such as concurrence, quantum discord (QD) and entanglement entropy. These analyses are carried out on finite clusters as shown in Fig. 1.

The results of our investigations reveal several interesting insights. First, we find that frustration does not induce entanglement between pairs of spins in next-nearest neighbour (NNN) positions. Conversely, entanglement is observed within nearest neighbour (NN) spin pairs, particularly in an intermediate parameter range. This region is characterised by the presence of quantum discordance between both NN and NNN spin pairs. Through a careful study of the first derivative of the concordance and quantum discord, we successfully identify all three critical points in full agreement with previously reported fidelity results^28^. By partitioning the cluster into two distinct subsystems and exploiting the concept of entanglement entropy, our analysis provides further valuable insights. In particular, our observations strongly suggest that the intermediate region is characterised by quantum entanglement. This phenomenon lends significant weight to the proposition that the most likely phase within this region is the quantum spin liquid (QSL) phase, rather than the antiferromagnetic Ising phase. The culmination of our efforts underscores the power of using quantum entanglement concepts as a means of unravelling the complex landscape of strongly correlated systems, providing a fresh and illuminating perspective on their behaviour near critical points.

The structure of the paper is as follows: Section II: We begin by introducing the core framework of our study - the spin-1/2 frustrated antiferromagnetic *XY* model on the honeycomb lattice. The model incorporates interactions between spins positioned at both nearest and next-nearest neighbours, and provides a crucial foundation for our subsequent analyses. Section III: Here we provide a brief overview of the fundamental concepts that are central to our investigation. In particular, we consider the principles underlying concurrence, quantum discordance (QD) and entanglement entropy. These concepts are explored from the perspective of information theory, providing a coherent basis for their subsequent application in our study. Section IV: This section serves as the core of our presentation, where we present and interpret our results. We systematically present the results concerning the behaviour of concurrence, quantum discord and entanglement entropy over both nearest neighbour (NN) and next-nearest neighbour (NNN) pairs of spins. Our analyses are carefully framed in terms of the parameter $\alpha$, which encapsulates the degree of frustration. Section V: Finally, we offer a comprehensive summary that encapsulates the key findings of our study.

## Model

In our study we investigate the 2D spin-1/2 XY frustrated antiferromagnetic Heisenberg honeycomb model. The Hamiltonian of the model is explicitly defined as

1 $$\begin{aligned} H=J_1 \sum _{<i,j>} (S_i^x S_j^x+ S_i^yS_j^y)+J_2\sum _{\ll i,j\gg } (S_i^x S_j^x+ S_i^y S_j^y) \end{aligned}$$

Here, the symbols hold the following meanings: $S_i^{x(y)}$ refers to the in-plane component of the spin-1/2 operator associated with the *i*-th site; $J_1$ and $J_2$ denote the antiferromagnetic coupling constants between spins situated at nearest neighbor (NN) and next-nearest neighbor (NNN) positions, respectively. The summation indexes, indicated as $<i,j>$ and $\ll i,j\gg$, encapsulate summations over all NN and NNN spin pairs. The frustration parameter $\alpha$ is defined as $\alpha =\frac{J_2}{J_1}$.

The ground-state phase diagram of this model has attracted considerable attention and has been explored using various quantum techniques. In particular, in a 2011 study by Varney et al. the exact diagonalisation method was applied to finite clusters, revealing a rich set of phases as follows: (I) an in-plane Neel phase in the region $\alpha <\alpha _{c_1}\sim 0.21$, (II) a gapless quantum spin liquid (QSL) phase in the region $\alpha _{c_1}\sim 0.21<\alpha <\alpha _{c_2}\sim 0. 35$, (III) a collinear spin wave phase dominates the region $\alpha _{c_2}\sim 0.35<\alpha <\alpha _{c_3}\sim 1.32$, and finally (IV) a $120^{\circ }$ ordered phase takes over when $\alpha >\alpha _{c_3} \sim 1.32$. It’s worth noting that the nature of the system within the intermediate region $\alpha _{c_1}<\alpha <\alpha _{c_2}$ remains uncertain, and ongoing investigations are aimed at unravelling its properties.

In the following sections, we embark on a distinctive exploration using key observables from the realm of quantum information. In particular, we focus on the entropy of concurrence, quantum discord (QD) and entanglement - all of which have proven to be powerful tools for probing complex quantum systems^42–46^. Our overarching goal is multifaceted: we aim to unravel the existence of the previously outlined phases, to pinpoint the exact locations of critical points within the parameter space, and to engage in a thoughtful discourse on the exotic intermediate region. This investigation will be aided by the fascinating lens of many-body entanglement, which offers a fresh perspective on the intricate behaviour of the system in question.

## Quantum correlations

The fields of statistical mechanics, condensed matter physics and quantum information theory converge in their fascination with interacting quantum many-body systems. In particular, the concept of entanglement serves as a fundamental link between these disciplines^47–50^. In the context of a bipartite system, entanglement occurs when it becomes impossible to distinguish the state of each subsystem independently from the state of the composite system. This distinct phenomenon highlights the intricate way in which quantum effects are intertwined, allowing one subsystem to influence and control another. As a result, entanglement is a cornerstone for understanding a spectrum of quantum phenomena and harnessing them for manipulation.

Of particular note are exotic quantum states such as quantum spin liquids (QSL)^8,51^, topological phases^52–54^, and systems exhibiting many-body localisation^55^. These distinctive quantum correlations are crucial in delineating the unique properties of these states. Interestingly, recent advances in experimental capabilities have revealed the accessibility of entanglement in quantum many-body systems^56–59^. Several methods have emerged to quantify and measure quantum correlations, including concurrence, quantum discord (QD) and entanglement entropy. These quantifiers provide a toolbox for detecting and characterising many-body entanglement, thereby enriching our understanding of the complex interplay within quantum systems.

### Concurrence

In this section, we provide a brief overview of a valuable tool in the field of entanglement measurement: the concurrence. This quantifier, known as the entanglement monotone, finds application in scenarios involving mixed states of two spin-1/2 particles. Its formal definition is as follows^60^:

2 $$\begin{aligned} C_{ij}=C(\rho _{ij})=Max \{ 0, \lambda _{0}- \lambda _{1}- \lambda _{2}- \lambda _{3} \}, \end{aligned}$$

where $\lambda _{i}$’s are the eigenvalues of the reduced density matrix $R_{ij}=\sqrt{\rho _{ij} \tilde{\rho }_{ij}}$ in which $\tilde{\rho }$ is spin-flipped state $\rho$ and is written as

3 $$\begin{aligned} \tilde{\rho }_{ij}=(\sigma ^{y}_{i} \otimes \sigma ^{y}_{j})~ \rho ^{*\ }_{ij} (\sigma ^{y}_{i} \otimes \sigma ^{y}_{j}). \end{aligned}$$

For a pair of spin-1/2 particles located at sites *i* and *j*, the concurrence can be determined by the corresponding reduced density matrix $\rho _{ij}$,

4 $$\begin{aligned} \rho _{ij}=\left( \begin{array}{cccc} X_{ij}^{+} &{} 0 &{} 0 &{} F_{ij}^{*} \\ 0 &{} Y_{ij}^{+} &{} Z_{ij}^{*} &{} 0 \\ 0 &{} Z_{ij} &{} Y_{ij}^{-} &{} 0 \\ F_{ij} &{} 0 &{} 0 &{} X_{ij}^{-} \\ \end{array} \right) , \end{aligned}$$

where

$$\begin{aligned} X_{ij}^{+}= & {} \langle (1/2+S_{i}^{z})(1/2+S_{j}^{z})\rangle ,\quad Y_{ij}^{+}= \langle (1/2+S_{i}^{z})(1/2-S_{j}^{z})\rangle ,\\ Y_{ij}^{-}= & {} \langle (1/2-S_{i}^{z})(1/2+S_{j}^{z})\rangle ,\quad X_{ij}^{-}= \langle (1/2-S_{i}^{z})(1/2-S_{j}^{z})\rangle ,\\ Z_{ij}= & {} \langle S_i^{+}S_{j}^{-}\rangle ,\quad F_{ij}= \langle S_i^{+}S_{j}^{+}\rangle \end{aligned}$$

and $\langle ...\rangle$ represents the expectation value on the ground state of a quantum system. Finally, the concurrence is given by the following expression,

$$\begin{aligned} C_{i,j} = \max {\{0,2 (C_1,C_2)\}}, \quad \quad C_1=|Z_{ij}|-\sqrt{X_{ij}^{+}X_{ij}^{-}}, \quad \quad C_2=|F_{ij}|-\sqrt{Y_{ij}^{+}Y_{ij}^{-}}~. \end{aligned}$$

### Quantum discord

In information theory, mutual information is a metric that quantifies the interdependence between two random variables. It indicates how much information can be gained about one variable by knowing the other correlated variable.

For a comprehensive understanding of the quantum correlations inherent in a bipartite state that remain unexplored by concurrence, the calculation of quantum discord (QD) becomes relevant^61,62^. Quantum discord captures the mismatch between quantum and classical correlations. In essence, it quantifies the information content that can be obtained from quantum measurements, taking into account the influence of quantum states.

To illustrate the concept of quantum discordance, consider a pair of spins at positions *i* and *j*. In classical information theory, the joint entropy - which reflects the information that can be extracted from the simultaneous observation of $S_i$ and $S_j$ - is characterised as

5 $$\begin{aligned} H(i, j)=- \sum p(i, j) \log _2 p(i, j), \end{aligned}$$

where *p*(*i*, *j*) is the joint probability distribution, which characterized the total correlation between two spins $S_i$ and $S_j$. In addition, the conditional entropy is given by

6 $$\begin{aligned} H(i | j)= H(i, j) - H(j), \end{aligned}$$

now, the mutual information is expressed as

7 $$\begin{aligned} {{\mathscr {I}}}(i: j)= H(i) + H(j) - H(i, j), \end{aligned}$$

also, it can be written as

8 $$\begin{aligned} {{\mathscr {I}}}(i: j)= H(i) - H(i | j). \end{aligned}$$

In classical information theory, these two phrases are equivalent. In quantum information theory, the Shannon entropy and probability distribution are replaced by the Von-Neumann entropy and density matrix, respectively. Thus, the quantum mutual information for a bipartite quantum state $\rho _{ij}$ can be redefined as

9 $$\begin{aligned} {{\mathscr {I}}}(\rho _{ij})= S(\rho _{i}) + S(\rho _{j}) + \sum \limits _{\alpha = 0}^3 {{\lambda _\alpha }} \log ({\lambda _\alpha }), \end{aligned}$$

and

10 $$\begin{aligned} {{\mathscr {C}}}(\rho _{ij})= S(\rho _{i}) - S(\rho _{i} | \rho _{j}), \end{aligned}$$

is classical correlation. $S(\rho _{i} | \rho _{j})$ is a quantum generalization of the conditional entropy and should be measured over all possible states of the subsystem $S_j$. Unlike classical information, ${{\mathscr {I}}}(\rho _{ij})$ and ${{\mathscr {C}}}(\rho _{ij})$ are not the same, and the difference between them is so-called QD,

11 $$\begin{aligned} QD={{\mathscr {I}}}(\rho _{ij}) - {{\mathscr {C}}}(\rho _{ij}) \end{aligned}$$

The parameters needed to calculate the total and classical correlations are provided from the elements of the reduced density matrix. Details of calculations of the QD are presented in supplemental document.

**Figure 2:**
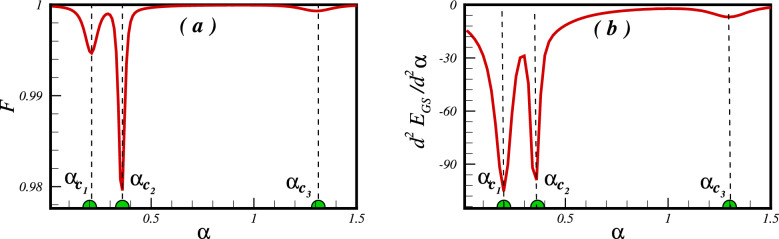
Signature of the quantum critical points is clearly seen in: (**a**) the fidelity metric as a function of frustration parameter $\alpha$ and (**b**) the second derivative of the ground state energy with respect to the frustration parameter for cluster with $N=24$ spins.

### Entanglement entropy

Entanglement entropy (EE) is a central concept defined as the von Neumann entropy of a reduced density matrix associated with a subsystem^63–67^. This measure quantifies the degree of correlation between two distinct subsystems, denoted *A* and *B*, within a composite quantum system. The EE is proving to be a powerful tool for characterising quantum phases imbued with many-body entanglement, and it finds application in revealing phase transitions in a wide range of lattice models relevant to condensed matter systems. Mathematically, the EE is expressed as

12 $$\begin{aligned} S_{A}=- Tr[ \rho _{A} log (\rho _{A})] \end{aligned}$$

Here $\rho _{A}= \text {Tr}{B} (\rho {AB})$ represents the reduced density matrix of the subsystem *A*, obtained by tracing over the complementary subsystem *B*. The EE obeys the area law, where for *d*-dimensional models it scales with the area of the subsystem *A* as $S_{A}= \alpha ~ l^{d-1}+...$, where the ellipses represent terms that decrease as the size *l* of the subsystem *A* tends to infinity. It is worth noting that the area law is strictly followed in non-critical systems, while critical systems show slight deviations due to multiplicative logarithmic corrections.

## Numerical results

In this section, we use two numerical techniques, namely the Lanczos^68^ numerical method and the Density Matrix Renormalisation Group (DMRG)^69^ method, to compute the ground state eigenvector of the system and to extract quantum correlations from honeycomb lattices of finite size. Notably, while the DMRG approach was originally developed for one-dimensional systems, its extension to two-dimensional geometries is challenging due to the exponential computational requirements arising from the width of the system^70^. Nevertheless, the lack of alternative methods (due to the limitations of quantum Monte Carlo for systems with the sign problem^71^ and the constraints of exact diagonalisation methods) positions the DMRG as a formidable tool for exploring the intricacies of complex two-dimensional systems^72–74^. The DMRG calculations detailed in this study were performed using the C++ library ITensor (version 3.1)^75^. Honeycomb lattices with finite clusters were treated under periodic boundary conditions. In particular, the focus was predominantly on symmetric hexagonal clusters (shown in Fig. 1) due to their tendency to reduce finite size effects. Supplementary investigations were carried out with clusters of different shapes, the results of which are presented in the supplementary document.

As the main focus of this study is to validate the results of Ref.^28^, it is appropriate to reproduce their results using the quantum ground state fidelity $F= \langle \psi _{Gs} (\alpha )| \psi _{Gs} (\alpha +\delta \alpha )| \rangle$, together with the second derivative of the ground state energy with respect to the frustration parameter. Figure 2 shows the results obtained by both the Lanczos and DMRG techniques for a cluster of $N=24$ spins. It is clear from the figure that the fidelity remains largely close to unity, except in the vicinity of the quantum critical points where it experiences a drop. This pattern is mirrored in the plot of the second derivative of the ground state energy, a quantity that aims to capture the characteristics of second order quantum phase transitions. In particular, the critical points are identified as $\alpha _{c_1}=0.214\pm 0.002$, $\alpha _{c_2}=0.352\pm 0.002$, $\alpha _{c_3}=1.272\pm 0.02$, thus achieving a robust agreement with both numerical and analytical methods. This congruence further strengthens the credibility of the approach adopted.

**Figure 3:**
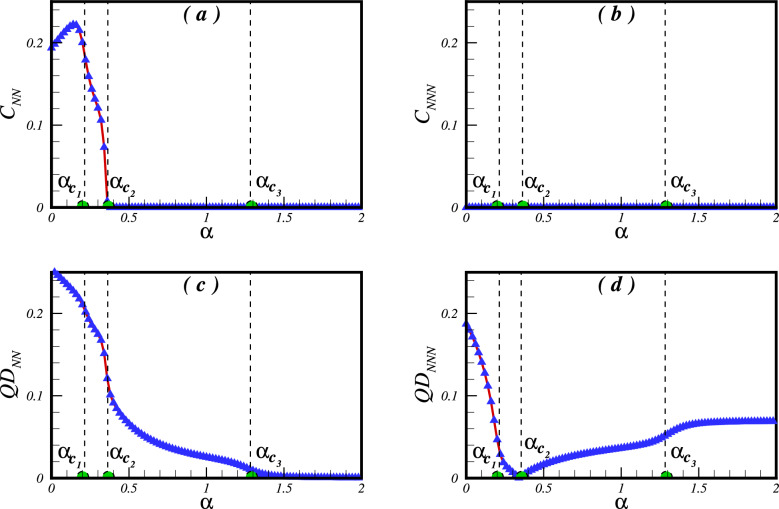
The concurrence and the QD as a function of the frustrated parameter $\alpha$ between the NN (**a**,**c**) and the NNN (**b**,**d**) pair of spins in a cluster of $N=24$ spins.

In Fig. 3, we demonstrate the complex behaviour of the concurrence and quantum discord (QD) between a pair of spins as they evolve with the frustration parameter $\alpha$, using a cluster of $N=24$. Figure 3a provides insight into the entanglement dynamics of the nearest-neighbour (NN) pair of spins across various degrees of frustration. Initially, in the absence of frustration, the NN pair is entangled. Upon the introduction of interaction between the next-nearest neighbour (NNN) pairs of spins, $C_{NN}$ displays growth until it reaches a value that is close to the first critical point - an indicative boundary of the quantum spin-liquid (QSL) phase. This trend illustrates how weak frustration amplifies quantum fluctuations, thereby intensifying the entanglement between NN pair spins through a mechanism of enhanced mixing. Subsequently, as frustration increases, the concurrence $C_{NN}$ decreases within the critical region. Upon entering the quantum spin liquid (QSL) phase, this concurrence experiences a monotonous decline, ultimately vanishing at the second critical point $\alpha _{c_2}$-a delineating threshold for the collinear spin-wave phase. Consequently, within the collinear and $120^{\circ }$ ordered phases, the nearest neighbour pair of spins do not exhibit entanglement.

The entanglement between the NN pair of spins is influenced by the expansion of interaction networks. In situations devoid of frustration, a particle interacts with three others whereas the introduction of frustration significantly increases this to nine interactions per particle. Due to the heightened interaction complexity, quantum fluctuations reduce, which causes a decline in entanglement, as observed in the examined model. Conversely, Fig. 3b shows that the entanglement between the next-nearest neighbor (NNN) pair of spins remains unaltered by the frustration parameter. This feature indicates the complex interaction among distinct areas of the phase diagram concerning entanglement formation and spread.

A better understanding of the intermediate phase within the region $\alpha _{c_1}<\alpha <\alpha _{c_2}$ can be achieved by examining the creation of valence-bond states, also known as singlet states, between pairs of spins. It has been established that a resonating valence-bond liquid can be depicted through a wave function that is a composition of numerous valence-bond configurations. Here, we have examined various valence-bond state setups among NN, NNN, and NNNN spin pairs and computed the corresponding valence-bond state parameters.

$$\begin{aligned} D=\frac{2}{N} \sum _{<i,j>} \langle \vec {S_i}. \vec {S_j} \rangle ,\qquad D^{'}=\frac{2}{N} \sum _{\ll i,j\gg } \langle \vec {S_i}. \vec {S_j} \rangle ,\qquad D^{''}=\frac{2}{N} \sum _{\lll i,j\ggg } \langle \vec {S_i}. \vec {S_j} \rangle . \end{aligned}$$

**Figure 4:**
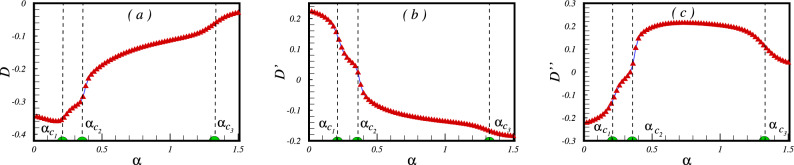
The valence bond parameter as a function of the frustrated parameter $\alpha$ between (**a**) the NN (**b**) the NNN pair of spins in a cluster of $N=24$ spins.

The numerical results are presented in Fig. 4. It is noteworthy that specific regions of the valence-bond state parameters, namely *D* and $D''$, offer significant insights into the underlying spin configurations. Negative values are observed for *D* and $D''$ in the region where $\alpha$ is less than $\alpha _{c_1}$. This negativity indicates the existence of Néel order, aligning precisely with the expected outcome in this phase. Interestingly, the intermediate region delineated by $\alpha _{c_1}<\alpha <\alpha _{c_2}$ exhibits unique behaviour. In this regime, both *D* and $D''$ take on negative values, with certain ranges of about $-0.35<D<-0.3$ and $-0.12<D''<0$. This distinct pattern strongly implies the formation of valence-bond states between neighbouring and second-nearest neighbouring pairs of spins - a definitive indication of the existence of a spin liquid phase within this middle region. To summarise, the complete examination of the valence-bond state parameters, as illustrated in Fig. 4, confirms the presence of distinct phases in the system. The negative and positive values of *D*, $D''$, and $D'$ provide informative indications of Néel order, spin liquid, and collinear spin-wave phases, respectively. This perceptive analysis enhances our comprehension of the complex behaviours displayed by this quantum system.

The quantum discord (QD) is a significant measure investigated in this context, and Fig. 3c,d illustrates its behaviour for the considered pair of spins. Notably, the QD is observed both between nearest-neighbor (NN) and next-nearest-neighbor (NNN) spins even in the absence of frustration ($\alpha =0$). This showcases the existence of quantum correlations even in the unfrustrated scenario. When frustration is introduced, intriguing trends emerge. Within the Néel phase, with increasing values of the frustration parameter $\alpha$, there is a decline in the quantum discord (QD) between nearest neighbour (NN) and next nearest neighbour (NNN) pairs of spins. Notably, the QD between NNN spins decreases at a higher rate than that between NN spins.

As the QD passes the first critical point $\alpha _{c_1}$, the behaviour between NN and NNN pairs becomes less distinct. Yet, at the second critical point $\alpha _{c_2}$, there is a noticeable drop in $\textrm{QD}_{\textrm{NN}}$, indicating substantial changes in the quantum correlations within the system. Meanwhile, $\textrm{QD}_{\textrm{NNN}}$ is almost negligible around this second critical point. Transitioning into the collinear spin-wave phase, we observe a notable change in behaviour between $\textrm{QD}_{\textrm{NN}}$ and $\textrm{QD}_{\textrm{NNN}}$. With increasing frustration parameter $\alpha$, the QD between NN spins decreases, suggesting a loss of quantum correlations. By contrast, $\textrm{QD}_{\textrm{NNN}}$ exhibits an increasing trend, indicating the emergence of quantum correlations among the NNN spins. This phenomenon can be understood by analysing the stabilization of the magnetic spin-wave structure. The structure requires compensating correlations amongst the nearest spins to sustain itself, where NNN spins, or second neighbours in the model, facilitate this compensation. Astonishingly, as the frustration parameter $\alpha$ increases, quantum correlations in the form of QD develop inversely between NN and NNN spins. Finally, upon reaching the third critical point $\alpha _{c_3}$, $\textrm{QD}_{\textrm{NN}}$ approaches zero and displays asymptotic behavior within the $120^{\circ }$ ordered phase. In contrast, $\textrm{QD}_{\textrm{NNN}}$ reaches a finite saturation value and remains relatively constant throughout the $120^{\circ }$ ordered phase. A brief explanation based on the reduced density matrix formalism for a three-spin toy model is provided in the supplementary document.

**Figure 5:**
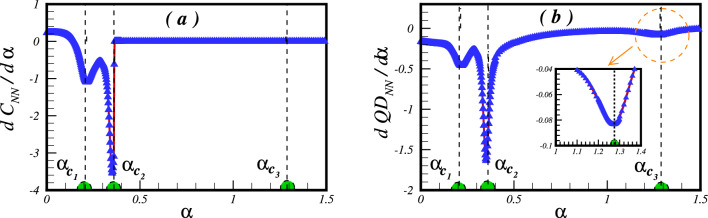
The first derivative of (**a**) the concurrence and (**b**) the QD between NN pair of spins with respect to the frustration parameter $\alpha$.

While quantum phase transitions are indicative of sudden changes in the ground state of a many-body system, the analysis of observables can offer significant insights into these transitions. In our study, we have calculated the first derivative of the concordance and quantum discordance (QD) between the nearest-neighbour (NN) spin pairs, as shown in Fig. 5. Remarkably, the significant drops seen in the first derivative of concurrence and QD for the NN spins correspond exactly to the critical points of quantum phase transitions: $\alpha _{c_1}=0.214\pm 0.002$, $\alpha _{c_2}=0.352\pm 0.002$, and $\alpha _{c_3}=1.272\pm 0.02$. This indicates that these notable changes can be attributed to substantial alterations in the ground state structure as the system undergoes these transitions. Notably, these crucial points represent values at boundaries between different phases, including the Néel phase, the quantum spin liquid phase, the collinear spin-wave phase, and the $120^{\circ }$ ordered phase. The sudden changes in the first derivatives of the concurrence and QD act as distinct indicators of the underlying transformations that take place within the system during these quantum phase transitions. In regions away from these critical points, the behaviour of the first derivatives of the concurrence and QD remains relatively stable and constant. This stability in the first derivative values suggests that the ground state structure remains relatively unchanged and the system retains its existing phase. This observation confirms the efficacy of these first order derivatives in capturing the significant changes that occur during quantum phase transitions, and enhances our understanding of the complex behaviour of the system in response to varying levels of frustration.

**Figure 6:**
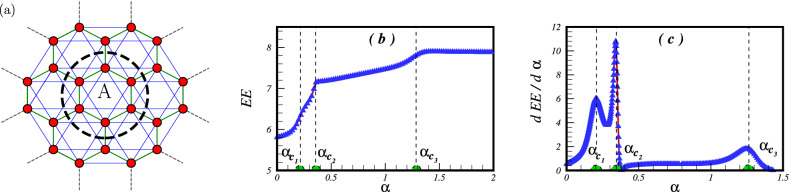
(**a**) The subsystem *A* as a hexagonal cell. (**b**) The EE versus the frustration parameter $\alpha$ for a cluster of $N=24$ spins. (**c**) The first derivative of the EE with respect to the frustration.

Our inquiry involved computing the entanglement entropy to reveal the complex patterns of entanglement within the system. To accomplish this, we partitioned the cluster of $N=24$ spins into two subsystems, designated A and B. In this arrangement, subsystem A represents the internal hexagonal cell, as depicted in Fig. 6a, while the findings of our investigation are exhibited in Fig. 6b. Initial observations based on Fig. 6b indicate that the inner hexagonal cell displays entanglement with the rest of the lattice in the absence of frustration. However, the introduction of frustration results in a noticeable rise in quantum correlations between this hexagonal cell and the surrounding system. This increase in entanglement is observed across different phases, which include the Néel phase, the quantum spin liquid (QSL) phase, and the collinear spin-wave phase. Notably, each phase displays different rates of entanglement growth. In contrast, the $120^{\circ }$ ordered phase shows a plateau-like behavior in the entanglement entropy. Although the first critical point does not exhibit a direct signature in the entanglement entropy, the locations of the second and third critical points are clearly distinguishable. Furthermore, calculations were conducted to determine the first derivative of the entanglement entropy regarding the frustration parameter. The results of this analysis are presented in Fig. 6c, showing the clear patterns of all critical points in the first derivative of the entanglement entropy. This confirms the usefulness of the entanglement entropy and its derivative as indicators of critical quantum phase transitions within the system. These findings add to our overall understanding of the intricate quantum correlations in the system’s behaviour.

## Conclusion

The investigation of the spin-1/2 anisotropic *XY* antiferromagnetic Heisenberg honeycomb model has posed an intriguing challenge in the world of low-dimensional magnets. At zero temperature, the model initially exhibits the expected long-range N’eel order. The crucial query arises when taking into account the introduction of antiferromagnetic next-nearest-neighbour (NNN) interactions, a phenomenon known as frustration. This creates a fundamental issue: determining the phases triggered by this frustration. The literature presents a dichotomy of views. On the one hand, some studies using methods such as the Lanczos numerical technique, variational Monte Carlo, and extended path integral Monte Carlo simulations have acknowledged the presence of a quantum spin liquid (QSL) phase in the intermediate region of frustration. Conversely, studies based on the numerical density matrix renormalisation group (DMRG) method and series expansion methods suggest that an antiferromagnetic Ising phase dominates instead of the QSL. As a result of this ambiguity, our study aims to provide an alternative method to indirectly investigate the ordering of the ground state in this intermediate region.

The quantum spin liquid (QSL) phase is known for its strong quantum fluctuations that prevent magnetic ordering at zero temperature, and is expected to induce entanglement among the spin-1/2 particles. On the other hand, the antiferromagnetic Ising phase reduces entanglement between spins. Based on this distinction, our investigation focuses on quantum correlations, specifically quantum discord (QD) and entanglement entropy (EE). By utilizing the Lanczos exact diagonalization and DMRG techniques, we quantitatively calculate quantum correlations in various cluster configurations as a function of the frustration parameter.

Our findings illuminate a pattern of entanglement: nearest neighbor (NN) pairs of spins exhibit entanglement within the intermediate region, whereas next-nearest neighbor (NNN) pairs lack entanglement and remain unaffected by the introduction of frustration. Notably, all critical points derived from the first derivative of these quantum correlations with respect to the frustration parameter align precisely with previous outcomes reported in the literature. Moreover, our analysis unveils the persistence of quantum entanglement within the intermediate region, lending suport to the notion that the quantum spin-liquid (QSL) phase may hold stronger potential compared to the antiferromagnetic Ising phase within this parameter range. Nevertheless, we acknowledge that concerns persist regarding the existence of the Ising phase. Our study opens avenues for further exploration of spin-1/2 2D models by harnessing the power of quantum information techniques, particularly entanglement and quantum discord (QD), to gain deeper insights into the intricate nature of quantum phase transitions and emergent phases in condensed matter systems.

### Supplementary Information

Supplementary Information.

## Data Availability

The data sets used and/or analysed during the current study available from the corresponding author on reasonable request.
