# Supplementary material for: Quantum correlations in the frustrated XY model on the honeycomb lattice
Source: Sci Rep. 2023 Sep 25;13:16034. doi: 10.1038/s41598-023-43080-3 (PMC10520029; doi:10.1038/s41598-023-43080-3)
Supplement: Supplementary file 1 — Supplementary Information. [file 41598_2023_43080_MOESM1_ESM.pdf]

# Quantum correlations in the frustrated XY model on the honeycomb lattice: Supplementary Material

Sahar Satoori,[1] Saeed Mahdavifar,[1,*] and Javad Vahedi[2,3,†]

[1]Department of Physics, University of Guilan, 45196-313, Rasht, Iran.
[2]Jacobs University, School of Engineering and Science, Campus Ring 1, 28759 Bremen, Germany.
[3]Department of Physics, Sari Branch, Islamic Azad University, 48161-19318, Sari, Iran.
(Dated: September 20, 2023)

## I. QUANTUM DISCORD

The QD between a pair of spins at sites $i$ and $j$ is defined as

$$QD_{i,j} = \mathcal{I}(\rho_{i,j}) - \mathcal{C}(\rho_{i,j}), \tag{1}$$

where the mutual information is given by

$$\mathcal{I}(\rho_{i,j}) = S(\rho_i) + S(\rho_j) + \sum_{\alpha=0}^{3} \lambda_\alpha \log(\lambda_\alpha). \tag{2}$$

$\lambda_\alpha$ are eigenvalues of $\rho_{i,j}$ and can be read as

$$\begin{aligned}
\lambda_1 &= \frac{1}{4}(1 + c_3 + \sqrt{4c_4^2 + (c_1 - c_2)^2}),\\
\lambda_2 &= \frac{1}{4}(1 + c_3 - (\sqrt{4c_4^2 + (c_1 - c_2)^2}),\\
\lambda_3 &= \frac{1}{4}(1 - c_3 + |c_1 + c_2|),\\
\lambda_4 &= \frac{1}{4}(1 - c_3 - |c_1 + c_2|),
\end{aligned} \tag{3}$$

and

$$\begin{aligned}
&S(\rho_i) = S(\rho_j) =\\
&\quad -\left[(\tfrac{1+c_4}{2})\log(\tfrac{1+c_4}{2}) + (\tfrac{1-c_4}{2})\log(\tfrac{1-c_4}{2})\right],
\end{aligned} \tag{4}$$

where we define new variables as

$$\begin{aligned}
c_1 &= 2Z_{i,j},\\
c_2 &= 2Z_{i,j},\\
c_3 &= X_{i,j}^{+} + X_{i,j}^{-} - Y_{i,j}^{+} - Y_{i,j}^{-},\\
c_4 &= X_{i,j}^{+} - X_{i,j}^{-}.
\end{aligned} \tag{5}$$

The classical correlations, $\mathcal{C}(\rho_{i,j})$, is given by

$$\begin{aligned}
&\mathcal{C}(\rho_{i,j}) =\\
&\max_{\{\Pi_i{}^B\}} \left(S(\rho_i) - \tfrac{S(\rho_0)+S(\rho_1)}{2} - c_4\cos(\theta)\tfrac{S(\rho_0)-S(\rho_1)}{2}\right),
\end{aligned} \tag{6}$$

where

$$\begin{aligned}
S(\rho_k) = &-\left(\tfrac{1+\theta_k}{2}\right)\log\left(\tfrac{1+\theta_k}{2}\right)\\
&-\left(\tfrac{1-\theta_k}{2}\right)\log\left(\tfrac{1-\theta_k}{2}\right),
\end{aligned} \tag{7}$$

and $\theta_k = \sqrt{\sum_{d=1}^{3} q_{kd}^2}$. In this equation $q_k$ is defined as

$$\begin{aligned}
q_{k1} &= (-1)^k c_1 \left[\frac{\sin\theta\cos\phi}{1 + (-1)^k c_4 \cos\theta}\right],\\
q_{k2} &= (-1)^k c_2 \left[\frac{\sin\theta\sin\phi}{1 + (-1)^k c_4 \cos\theta}\right],\\
q_{k3} &= (-1)^k \left[\frac{c_3\cos\theta + (-1)^k c_4}{1 + (-1)^k c_4 \cos\theta}\right].
\end{aligned} \tag{8}$$

where $0 \le \theta \le \pi$ and $0 \le \phi \le 2\pi$ and $(j) = B$ is a set of projectors for a local measurement on part $j$.

## II. FINITE SIZE EFFECT

Here, we present the size effect on the numerical results. Different clusters with $N = 20, 22, 24, 26$ are shown in top panel of Fig. 1. Numerical ED results of the concurrence (Fig. 2 (a) and (b)) and the QD (Fig. 2 (c) and (d)) are presented. As is seen, extracted numerical on all sizes are in good agreement together and show the same behavior with respect to the frustration. We also present DMRG results for two clusters with size $N = 54, 96$, where we consider only the hexagonal geometry. As it can be seen, although DMRG results show some fluctuations in the QSL phase, but show a general trend compare with the ED results.

## III. ENTANGLEMENT IN $120^\circ$ ORDERED PHASE

In the limit of $\alpha \longrightarrow \infty$ where the system is decoupled to $\frac{N}{3}$ independent trimmers constructed of the NNN spins in the honeycomb lattice. The ground state of a trimmer is obtained as

$$|Gs\rangle_{tr} = \frac{1}{\sqrt{2}}[|\uparrow\uparrow\downarrow\rangle - |\uparrow\downarrow\uparrow\rangle]. \tag{9}$$

The reduced density matrix of a pair of spins in the pure $120^\circ$ ordered phase is simply find as

$$\rho_{ij} = \begin{pmatrix} \frac{1}{2} & 0 & 0 & 0 \\ 0 & \frac{1}{2} & 0 & 0 \\ 0 & 0 & 0 & 0 \\ 0 & 0 & 0 & 0 \end{pmatrix}, \tag{10}$$

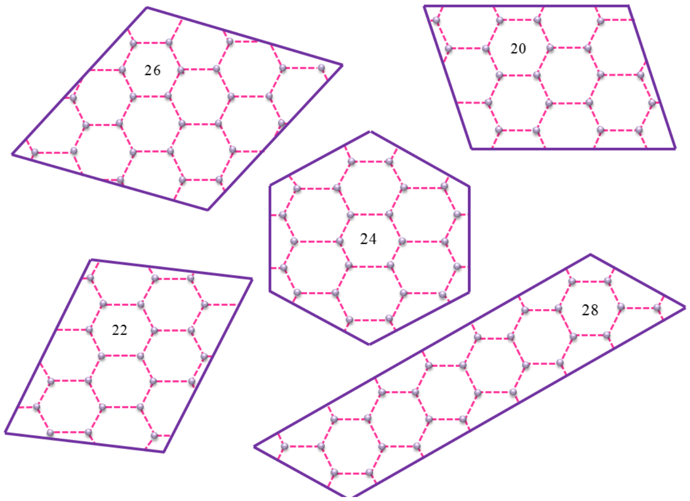


FIG. 1. Clusters used for finite size effect study in Fig.2.

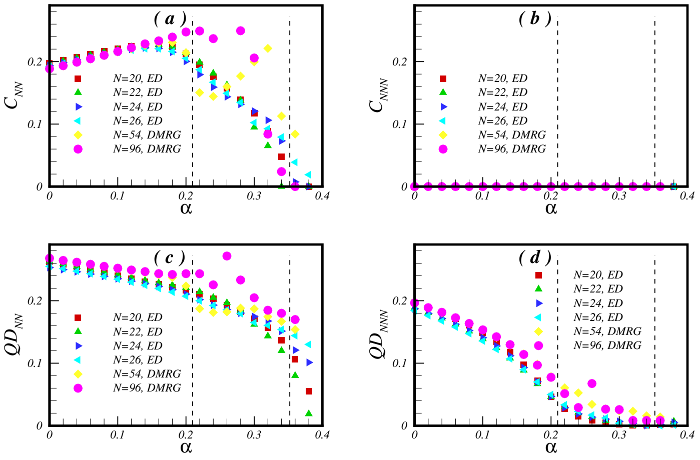


FIG. 2. Finite size study of the concurrence and the QD regrading clusters shown in Fig.1 between the NN ((a) and (c)) and the NNN ((b) and (d)) pair of spins for using ED and DMRG methods. Note that for the DMRG we consider the extension of hexagon-shaped cluster shown with $N = 24$ in Fig.1.

which gives zero entanglement between NNN pair of spins. On the other hand, since NN pair of spins are on independent trimmers, the reduced density matrix becomes

$$\rho_{ij} = \begin{pmatrix} 1 & 0 & 0 & 0 \\ 0 & 0 & 0 & 0 \\ 0 & 0 & 0 & 0 \\ 0 & 0 & 0 & 0 \end{pmatrix}, \tag{11}$$

which shows separability with no entanglement.
